# Supplementary material for: Combined Host- and Pathogen-Directed Therapy for the Control of Mycobacterium abscessus Infection
Source: Microbiol Spectr. 2022 Jan 26;10(1):e02546-21. doi: 10.1128/spectrum.02546-21 (PMC8791191; doi:10.1128/spectrum.02546-21)
Supplement: SUPPLEMENTAL FILE 1 — Supplemental material. Download SPECTRUM02546-21_Supp_1_seq3.pdf, PDF file, 2.3 MB [file spectrum02546-21_supp_1_seq3.pdf]

**Supplementary information**

# Combined host- and pathogen-directed therapy for the control of *Mycobacterium abscessus* infection

Noemi Poerio[1,a], Camilla Riva[2,a], Tommaso Olimpieri[1], Marco Rossi[2], Nicola I. Lorè[2], Federica De Santis[1], Lucia Henrici De Angelis[1], Fabiana Ciciriello[3], Marco M. D'Andrea[1], Vincenzina Lucidi[3], Daniela M. Cirillo[2,b], and Maurizio Fraziano[1,b #]

[1] Department of Biology, University of Rome "Tor Vergata"; Rome, Italy

[2] Emerging bacteria pathogens unit, San Raffaele Scientific Institute; Milan, Italy

[3] Department of Pediatric Medicine, Cystic Fibrosis Complex Operating Unit, Bambino Gesù Pediatric Hospital; Rome, Italy.

**# Corresponding author:**

Prof. Maurizio Fraziano,

Department of Biology

University of Rome "Tor Vergata"

Via della Ricerca Scientifica, 1 – 00133

Rome, Italy

Email: fraziano@bio.uniroma2.it

[a] Noemi Poerio and Camilla Riva contributed equally to this article. The author order was determined by their equal but gradated contributions for this paper.

[b] Maurizio Fraziano and Daniela M. Cirillo contributed equally to this article. The author order was determined by their equal but gradated contributions for this paper.

**Running title:** Combined therapy against *Mycobacterium abscessus*

**Abstract/Importance words count:** 227/143

**Text words count:** 2820

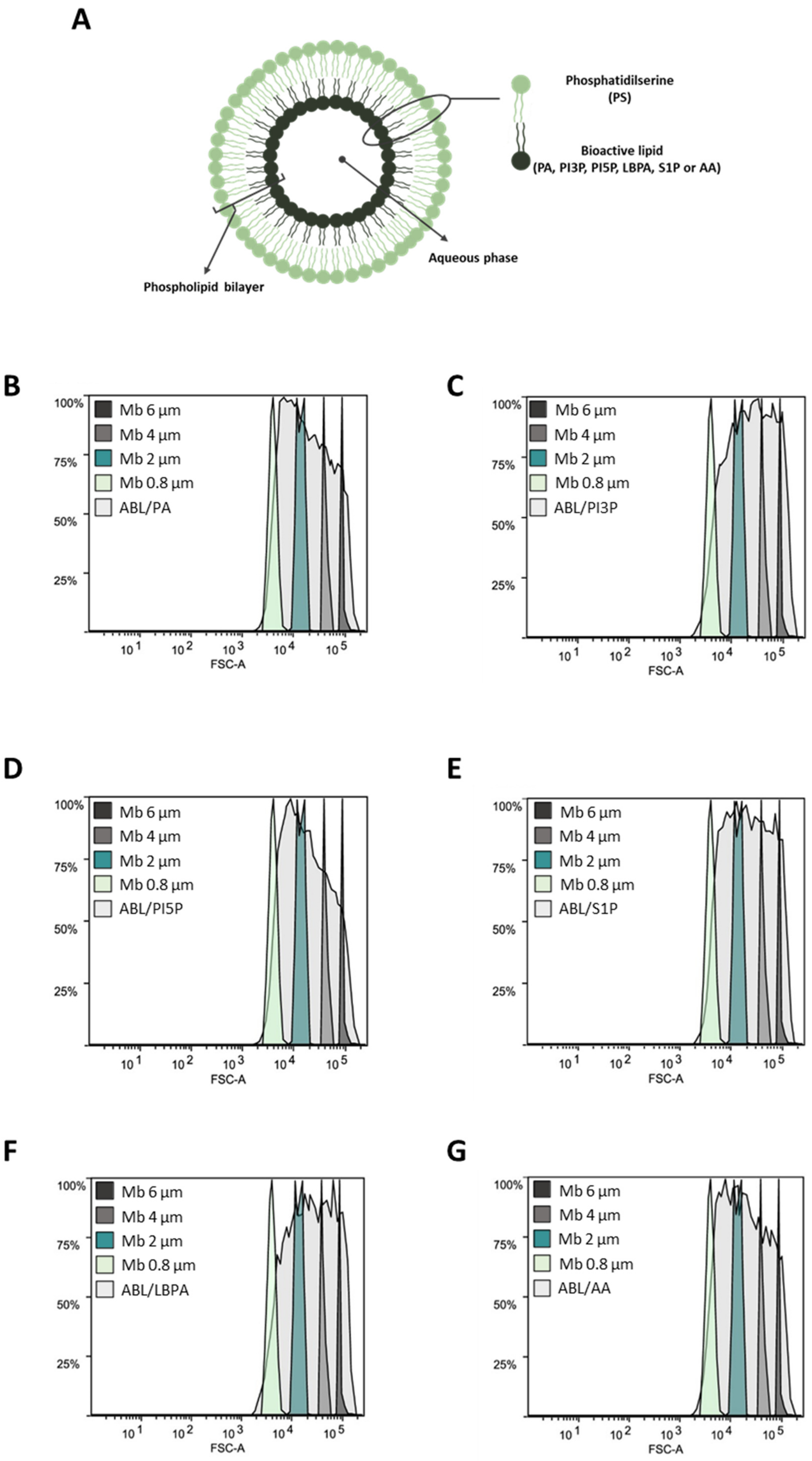
A
Phosphatidilserine
(PS)
Bioactive lipid
(PA, PI3P, PI5P, LBPA, S1P or AA)
Aqueous phase
Phospholipid bilayer
B
Mb 6 µm
Mb 4 µm
Mb 2 µm
Mb 0.8 µm
ABL/PA
C
ABL/PI3P
D
ABL/PI5P
E
ABL/S1P
F
ABL/LBPA
G
ABL/AA
100%
75%
50%
25%
10 1
10 2
10 3
10 4
10 5
FSC-A

S1

**S1. Graphic representation and dimensional analysis of ABLs.** (A) Representative drawing of an asymmetric liposome made of phosphatidylserine (PS) as the outer leaflet and a bioactive lipid as the inner leaflet (PA, phosphatidic acid; PI3P, phosphatidylinositol 3-phosphate, PI5P, phosphatidylinositol 5-phosphate; LBPA, lysobisphosphatidic acid (LBPA), S1P, sphingosine 1-phosphate; AA, arachidonic acid). The average diameter of ABL was assessed comparing the median forward scatter (FS) of (B) ABL/PA, (C) ABL/PI3P, (D) ABL/PI5P, (E) ABL/S1P, (F) ABL/LBPA, (G) ABL/AA with that of commercially available microbeads (Mb) with known diameters (0.8 - Sigma, 2, 4 and 6 µm - Thermo Fisher), acquired on a Celesta flow cytometer and analysed by the FlowLogic software (Miltenybiotec). Each sample is displayed as a proportion of its highest value, which is shown at 100% (according to FlowLogic user's manual).

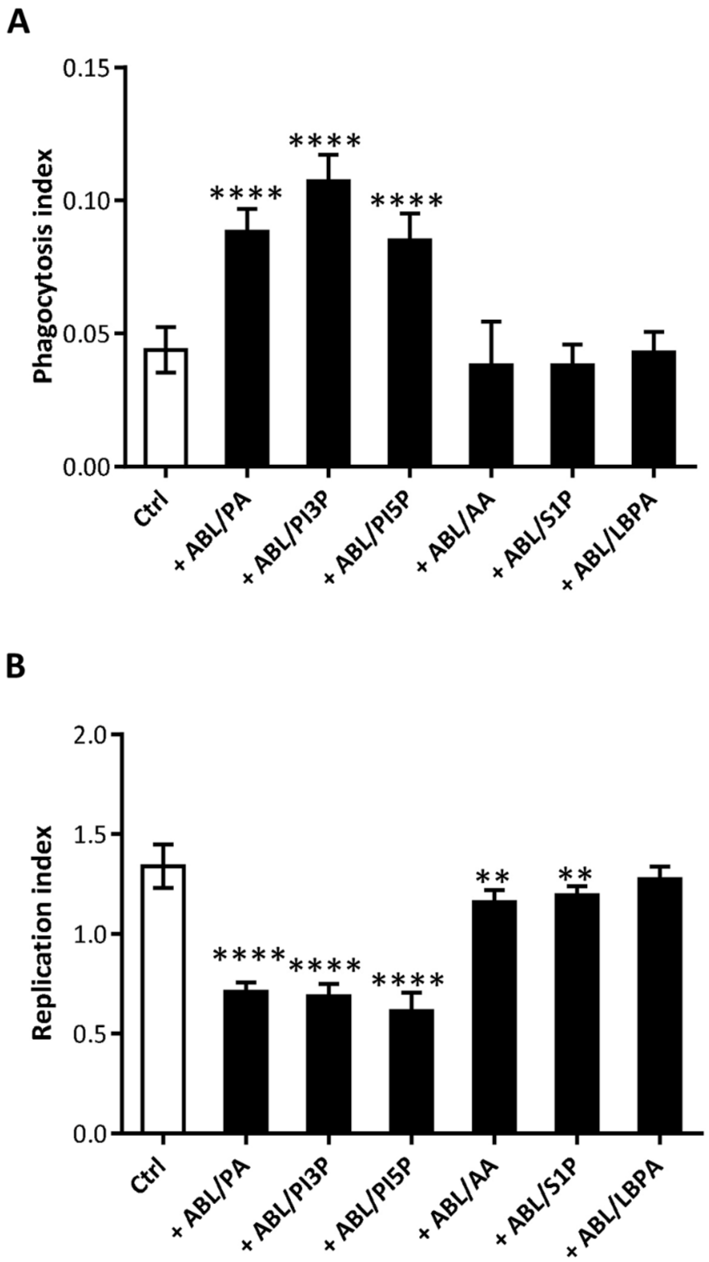
A
Phagocytosis index
0.15
0.10
0.05
0.00
****
****
****
Ctrl
+ ABL/PA
+ ABL/PI3P
+ ABL/PI5P
+ ABL/AA
+ ABL/S1P
+ ABL/LBPA
B
Replication index
2.0
1.5
1.0
0.5
0.0
****
****
****
**
**
Ctrl
+ ABL/PA
+ ABL/PI3P
+ ABL/PI5P
+ ABL/AA
+ ABL/S1P
+ ABL/LBPA

**S2. ABL/PA, ABL/PI3P and ABL/PI5P enhance both internalization and intracellular killing of Mab in dTHP-1 cells.** dTHP-1 cells, were cultured at $5x10^5$ cells/well in 24 well plates and $2x10^5$ cells/well in 96 well plates, respectively. Cells were stimulated with selected ABL formulations before infection for 30 min, and then infected with Mab reference strain (ATCC 19977). (A) The bacterial uptake was quantified by CFU assay and indicated as phagocytosis index, calculated as the ratio between the CFUs obtained immediately after the infection and the inoculum. Cells were exposed or not to INH172, infected with Mab and treated for 18 hours with the selected ABL formulations. (B) Bacterial growth was assessed by CFU assay. Replication index was calculated as the ratio between the CFU obtained after 18 hours from infection, in the presence or absence of ABL formulations, and the CFU obtained before the addition of liposomes. The results are shown as mean ± standard deviation of the values obtained from triplicate of each condition. **$p<0.01$, **** $p< 0.0001$ by Student's test.

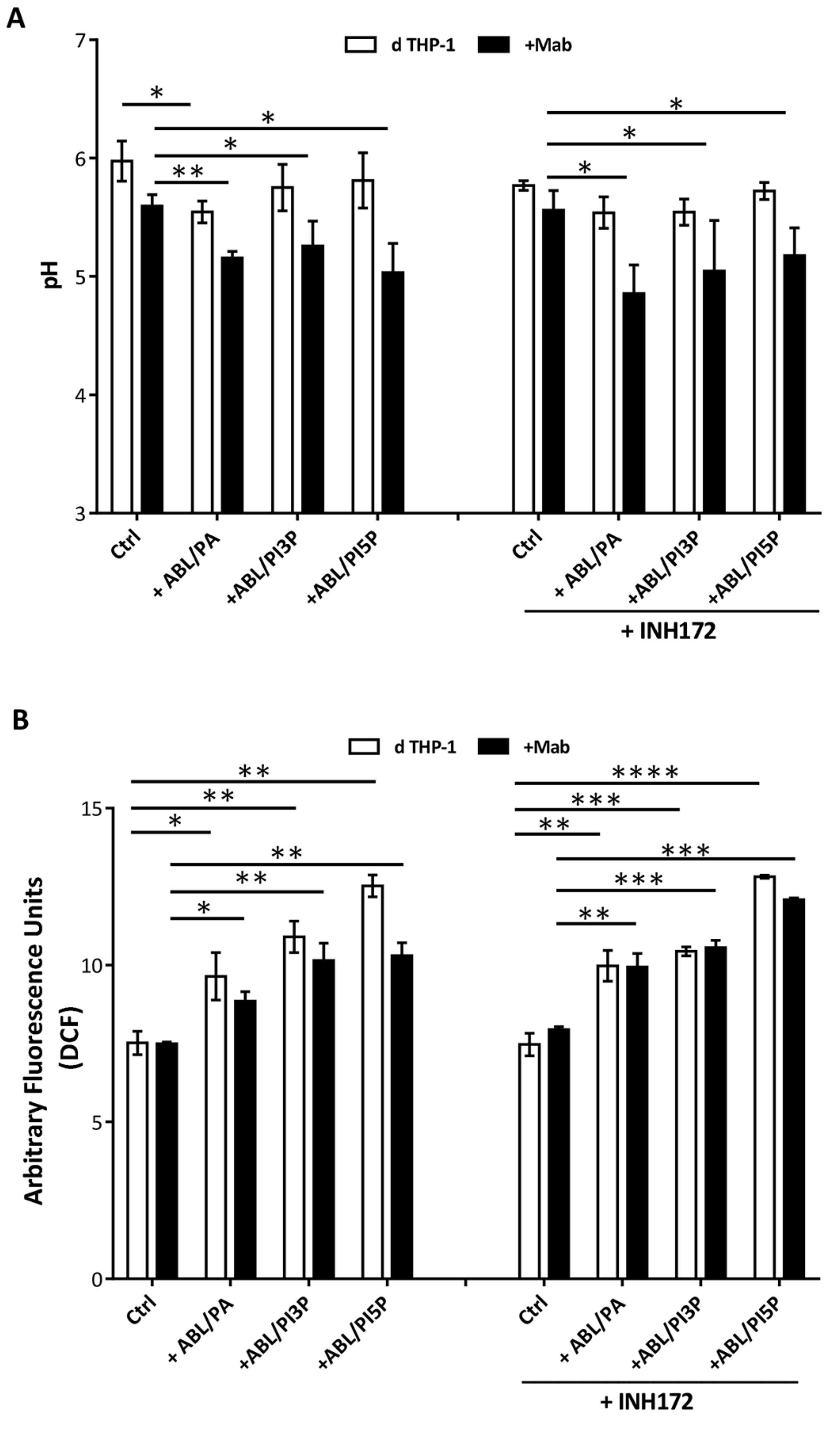
A
d THP-1
+Mab
pH
7
6
5
4
3
Ctrl
+ ABL/PA
+ABL/PI3P
+ABL/PI5P
+ INH172
B
Arbitrary Fluorescence Units (DCF)
15
10
5
0


S3

**S3. Selected ABL formulations promote phagosome acidification and ROS production in human macrophages treated or not with INH172 and infected or not with Mab.** dTHP-1 cells, treated or not with INH172, were infected or not with Mab and stimulated with ABL/PA, ABL/PI3P or ABL/PI5P for 3 hours. Phagosome pH (A) and ROS (B) were assessed, by staining cells with Lysosensor green DND 189 and DCF, respectively. Results are shown as mean ± standard deviation of the values obtained from triplicate cultures and are representative of two different experiments. * p< 0,05; ** p< 0,01, ***p< 0,001, ****p< 0,0001 in comparison with untreated cells by one-sided Student's t test.

A

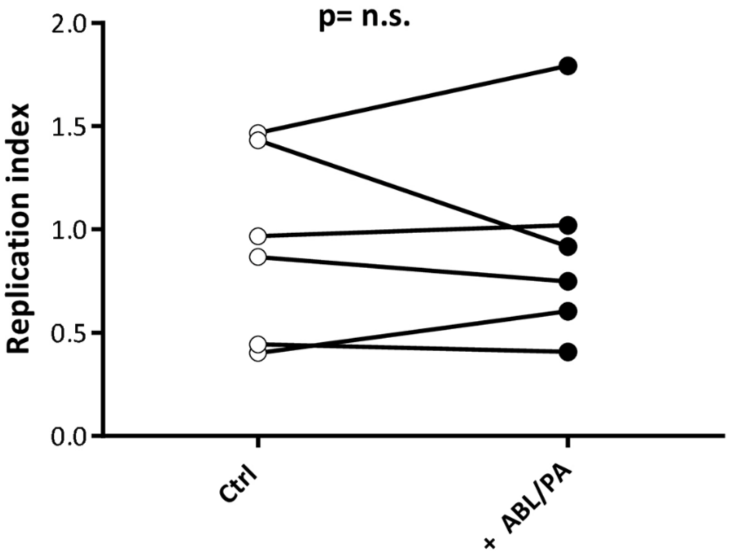
p= n.s.
Replication index
2.0
1.5
1.0
0.5
0.0
Ctrl
+ ABL/PA

B

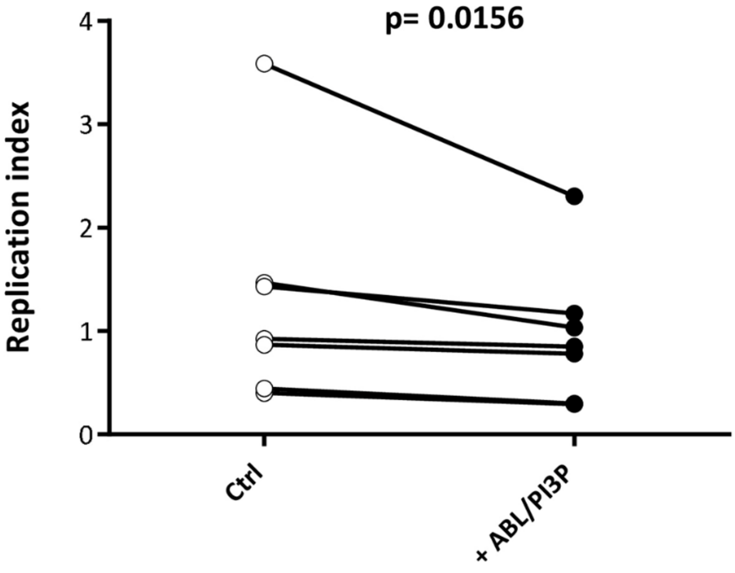
p= 0.0156
Replication index
4
3
2
1
0
Ctrl
+ ABL/PI3P

**S4. ABL/PI3P but not ABL/PA promotes intracellular Mab killing in CF MDM.** MDM isolated from CF patients (n=8), were plated at the concentration of $1x10^6$ cells/ml, infected with Mab reference strain (ATCC 19977) and then stimulated for 18 hours with ABL carrying PA (A , n=6) or PI3P (B, n= 7). Bacterial growth was assessed by CFU assay and replication index was calculated as the ratio between the CFU obtained after 18 hours from infection, in the presence or absence of ABL formulations, and the CFU obtained before the addition of liposomes. Statistical analysis was performed by using two-sided Wilcoxon matched-pairs signed rank test. (A) p=not significant (n.s.); (B) p=0.0156.

# 8 days of treatment

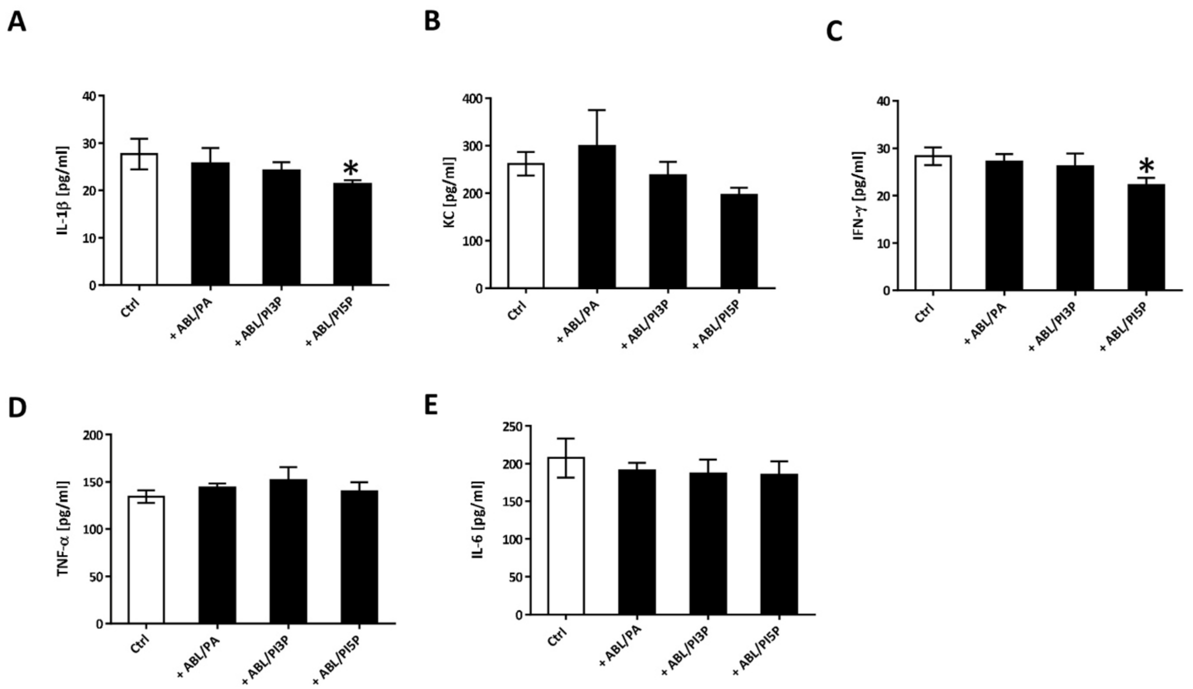


# 29 days of treatment

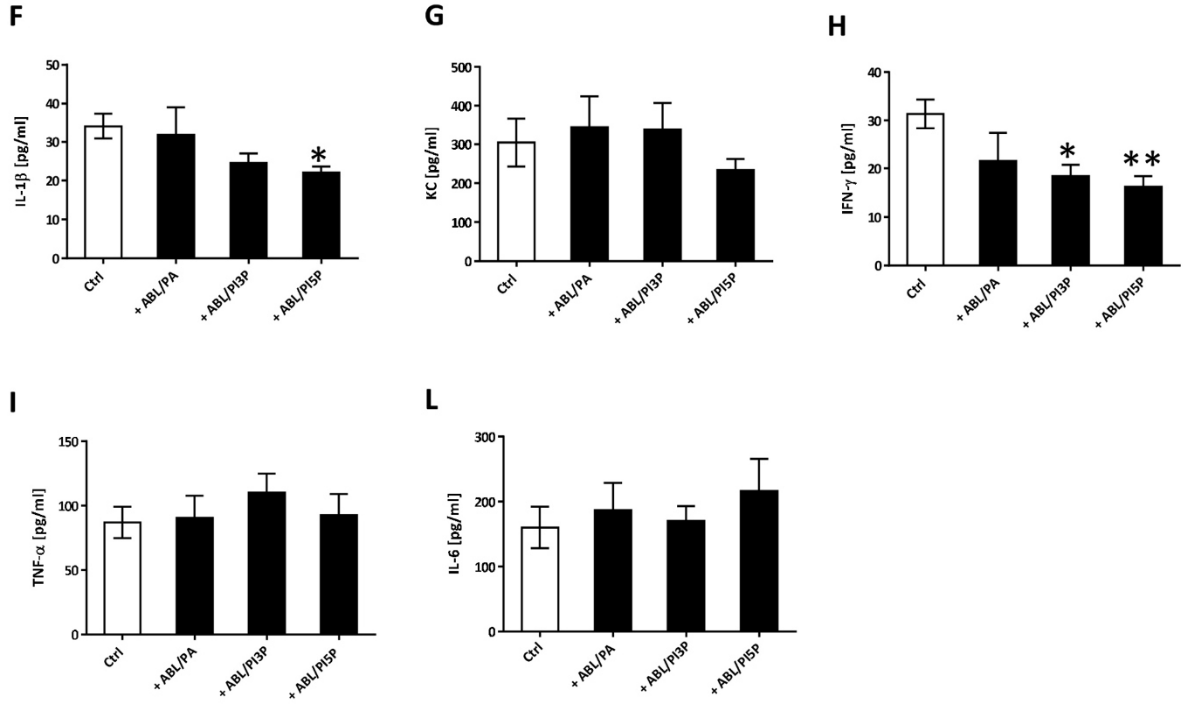

**S5. ABL/PI5P down-modulate IL-1β and IFN-γ release in *in vivo* model of Mab infection.** C57BL/6N (Charles River) mice were chronically infected by i.t. injection with $10^5$ CFU of Mab (reference strain ATCC 19977) and treated with ABL/PA, ABL/PI3P and ABL/PI5P, 3 times a week, starting from day 7 after infection. IL-1β, KC, IFN-γ, TNF-α and IL- 6 were evaluated in the mice lung at day 15 (A) and 36 (B) after infection. The data were pooled from two independent experiments. The results are shown as median of the values and statistical significance was evaluated by Mann Whitney test (* $p<0.05$, ** $p<0.01$, *** $p<0.001$).

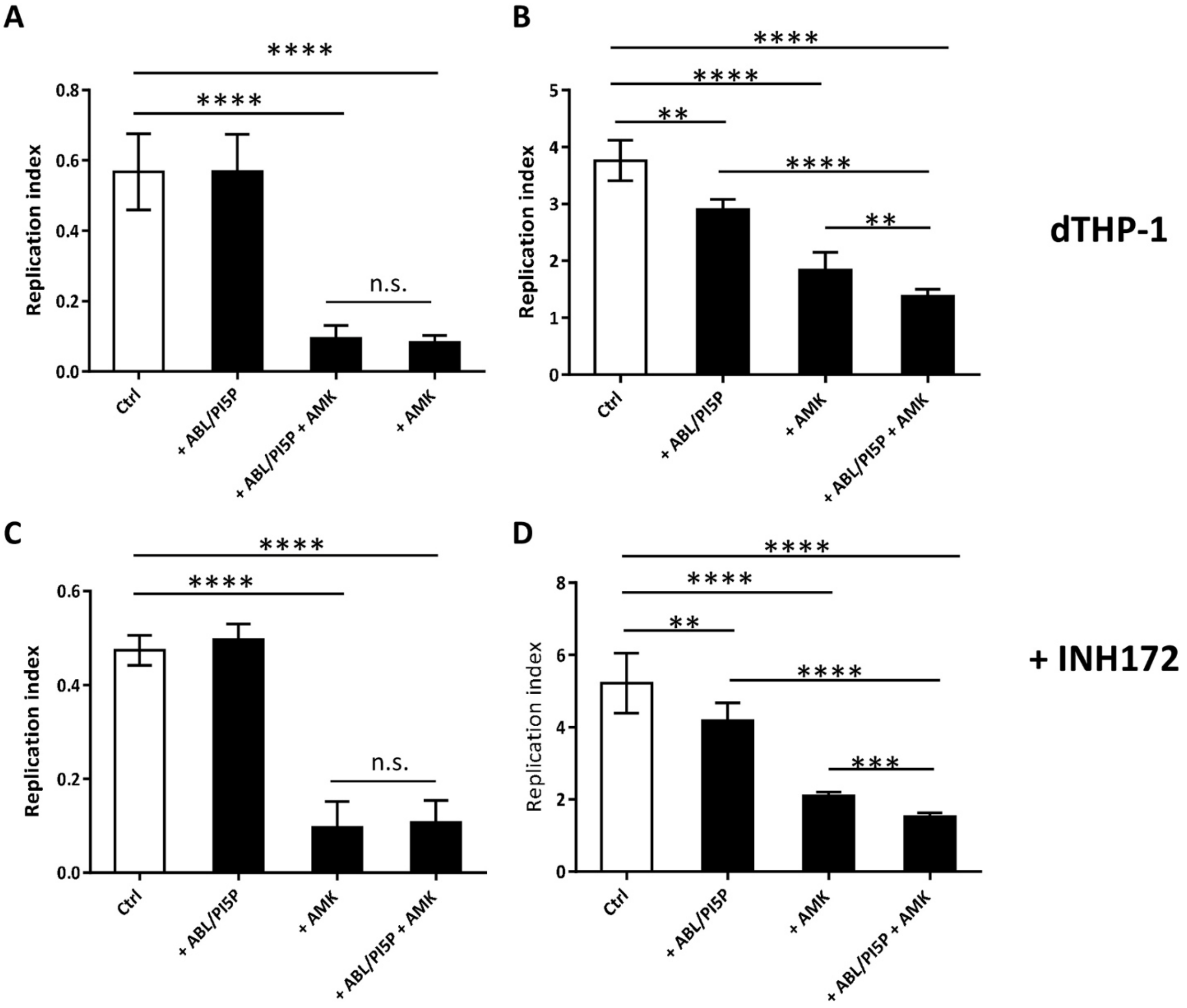
A
B
C
D
Replication index
Ctrl
+ ABL/PI5P
+ ABL/PI5P + AMK
+ AMK
n.s.
****
**
***
dTHP-1
+ INH172

**S6. ABL/PI5P-amikacin combined treatment promotes higher reduction intracellular growth of Mab than single treatments.** dTHP-1 cells were cultured at the concentration of $5x10^5$ cells/ml, exposed (C and D) or not (A and B) to INH172 and infected with Mab. Cells were then stimulated, with ABL/PI5P and/or 4 µg/ml amikacin (AMK) for further 18 hours. Finally, supernatant was collected, cells were lysed and both analysed for extracellular (A and C) and intracellular (B and D) bacterial growth, respectively. Replication index was calculated as the ratio between the CFU obtained 18 hours after infection in the presence or absence of ABL/PI5P and/or AMK, and those obtained at time 0 immediately after infection and before the addition of the stimuli. The results are shown as mean ± standard deviation of the values obtained from triplicate of each condition and are representative of 3 different experiments. n.s. = not significant, **$p<0.01$, *** $p<0.001$ and **** $p< 0.0001$ by Student's t test.

A

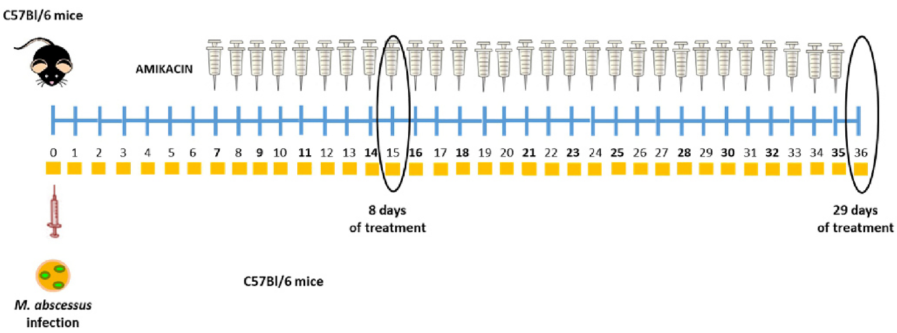
C57Bl/6 mice
AMIKACIN
0 1 2 3 4 5 6 7 8 9 10 11 12 13 14 15 16 17 18 19 20 21 22 23 24 25 26 27 28 29 30 31 32 33 34 35 36
8 days of treatment
29 days of treatment
M. abscessus infection
C57Bl/6 mice

B

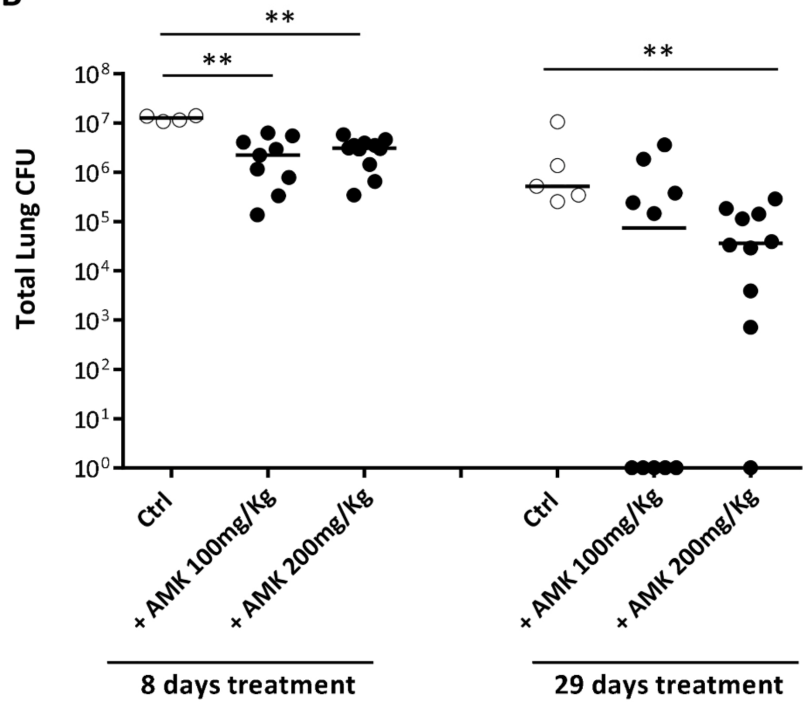
**
**
**
Total Lung CFU
$10^8$
$10^7$
$10^6$
$10^5$
$10^4$
$10^3$
$10^2$
$10^1$
$10^0$
Ctrl
+ AMK 100mg/Kg
+ AMK 200mg/Kg
Ctrl
+ AMK 100mg/Kg
+ AMK 200mg/Kg
8 days treatment
29 days treatment

**S7. Selection of Amikacin dose for next combination therapy.** C57BL/6N mice were chronically infected with $10^5$ Mab (reference strain ATCC 19977) by i.t. injection and treated with 100 mg/Kg or 200 mg/Kg of amikacin (AMK). AMK was administered every day starting from day 7 after infection by i.p. injection (A). At day 15 and 36 after infection mice were sacrificed, and lungs processed for the assessment of mycobacterial burden (B). The results are shown as median of the values and statistical significance by Mann Whitney test was performed (** $p<0.01$)

A

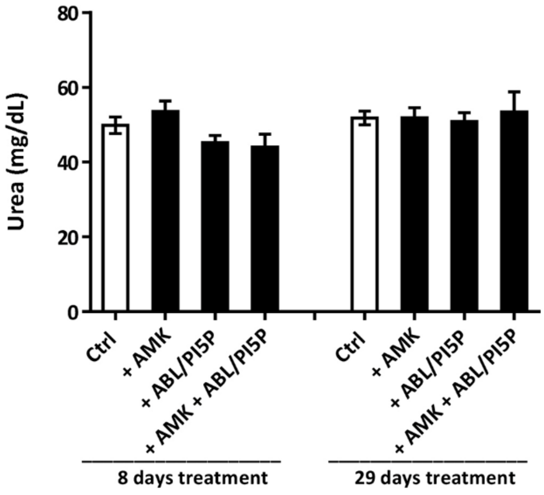

Urea (mg/dL)
80
60
40
20
0
Ctrl
+ AMK
+ ABL/PI5P
+ AMK + ABL/PI5P
Ctrl
+ AMK
+ ABL/PI5P
+ AMK + ABL/PI5P
8 days treatment
29 days treatment


B

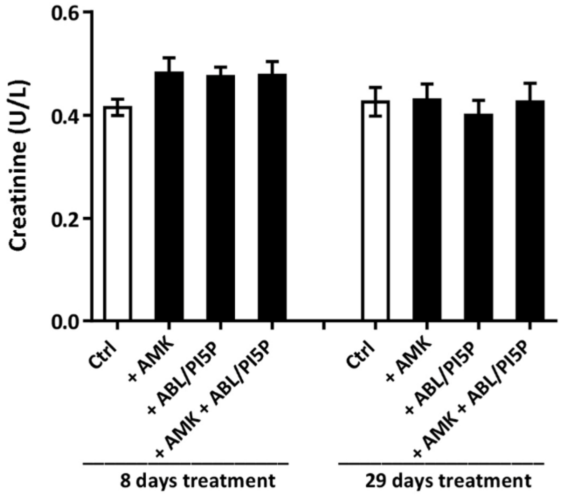

Creatinine (U/L)
0.6
0.4
0.2
0.0
Ctrl
+ AMK
+ ABL/PI5P
+ AMK + ABL/PI5P
Ctrl
+ AMK
+ ABL/PI5P
+ AMK + ABL/PI5P
8 days treatment
29 days treatment


C

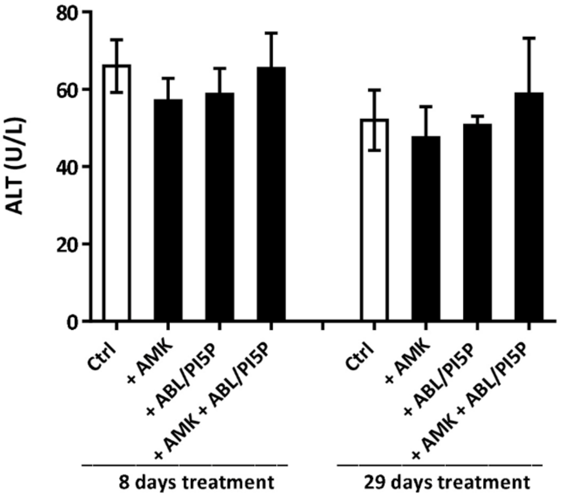

ALT (U/L)
80
60
40
20
0
Ctrl
+ AMK
+ ABL/PI5P
+ AMK + ABL/PI5P
Ctrl
+ AMK
+ ABL/PI5P
+ AMK + ABL/PI5P
8 days treatment
29 days treatment

**S8. Preliminary kidney and liver toxicity evaluation of ABL/PI5P-amikacin combination therapy.** C57BL/6N mice were intratracheally (i.t.) infected with $10^5$ CFU of Mab (reference strain ATCC 19977) and treated by i.p. injection with amikacin (AMK, 100 mg/Kg) and/or by i.n. administration of ABL/PI5P starting from day 7 after infection, according to the schedule indicated in Fig. 4C, and serum was drawn at day 15 and 36 for the assessment of Urea (A), creatinine (B) and Alanine aminotransferase (ALT) (C). The data were pooled from three independent experiments. The results are shown as median of the values and statistical significance by Mann Whitney test was performed.

# 8 days of treatment

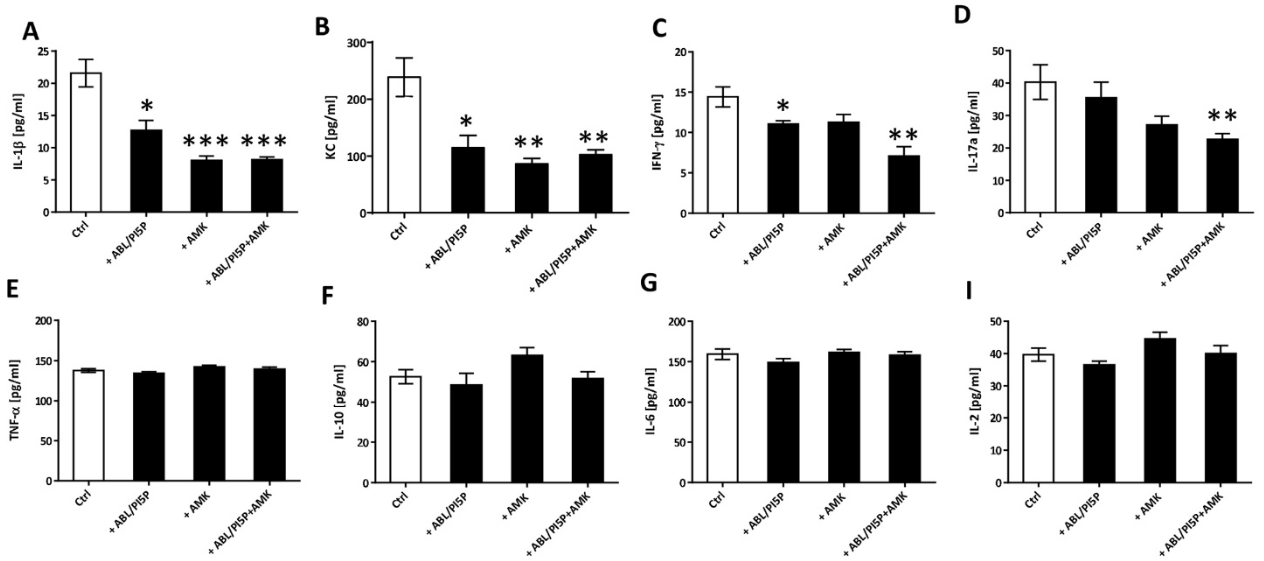


# 29 days of treatment

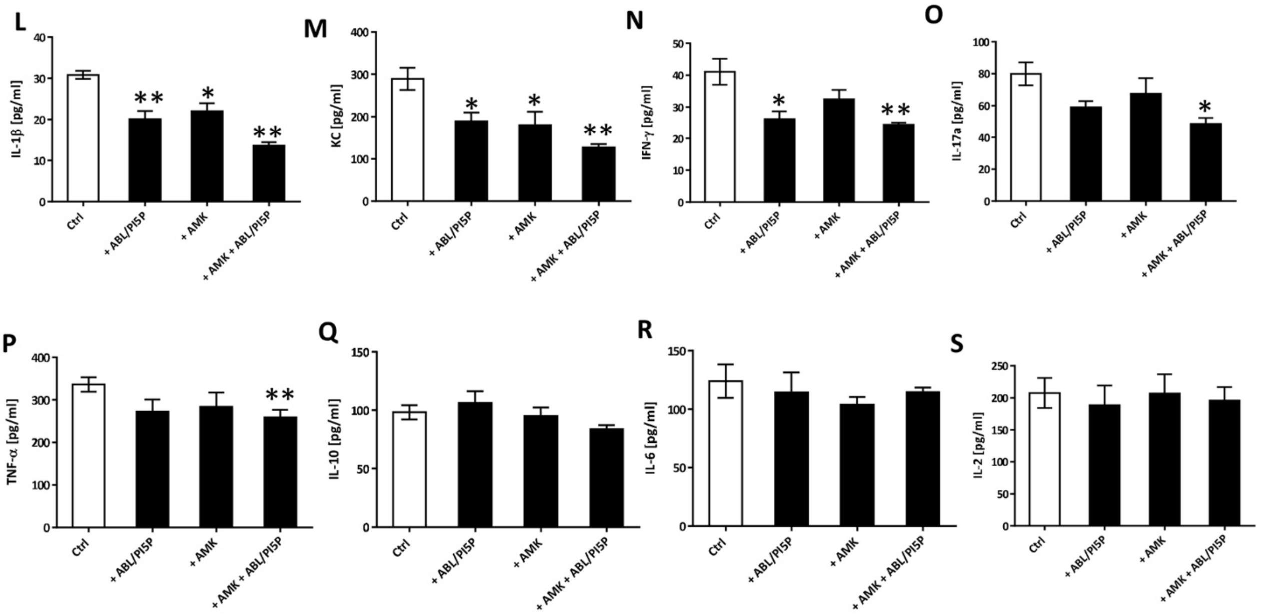

**S9.ABL/PI5P-amikacin combination treatment down-modulate proinflammatory cytokine release in an *in vivo* model of Mab infection.** C57BL/6N mice were intratracheally (i.t.) infected with $10^5$ CFU of Mab (reference strain ATCC 19977) and treated by i.p. injection with amikacin (AMK, 100 mg/Kg) and/or by i.n. administration of ABL/PI5P starting from day 7 after infection, according **to** the schedule indicated in Fig. 4C. IL-1β, KC, IFN-γ, IL-17a, TNF-α, IL-10, IL-2 and IL- 6 were evaluated in mice lung at day 15 (A-I) and 36 (L-S) after infection. The results are shown as median of the values and statistical significance by Mann Whitney test was performed. (* $p<0.05$, ** $p<0.01$, *** $p<0.001$).

**Supplementary table 1. Demographic and clinical characteristics of CF patients.**

| CF | Age (range) | Genotype | Microbiology | FEV-1 (%) |
|---|---|---|---|---|
| **1** | 30-34 | 1717-1G->A/DeltaF508 | *NTM, FF, MDR P.a.* | 57 |
| **2** | 21-25 | R347P/CFTRdele17a-18 | *P.a., MS P.a., MRSA, A.f.* | 91 |
| **3** | 7-11 | G542X/DeltaF508 | *A.x., NTM* | 55 |
| **4** | 25-29 | G542X/DeltaF508 | *MSSA, NTM* | 79 |
| **5** | 16-20 | G85E/DeltaF508 | *MSSA, P.a.* | 81 |
| **6** | 44-48 | DeltaF508/DeltaF508 | *MSSA, NTM, A.f.* | 60 |
| **7** | 22-26 | I1234V/DeltaF508 | *C.a., MDR P.a., E.f., NTM* | 57 |
| **8** | 36-40 | T5-TG13/DeltaF508 | *E.c., E.f.* | 60 |
| **9** | 23-27 | DeltaF508/DeltaF508 | *MS P.a., P.a., FF, C.a., A.f.* | 102 |
| **10** | 18-22 | DeltaF508/DeltaF508 | *MSSA* | 117 |
| **11** | 31-35 | L1077P/DeltaF508 | *MSSA, P.a., MDR P.a., FF* | 46 |
| **12** | 46-50 | A1006E/DeltaF508 | *A.x., P.a., MDR P.a., FF* | 54 |
| **13** | 33-37 | L1065P/DeltaF508 | *MSSA* | 101 |
| **14** | 36-40 | N1303K/W1282X | *MSSA, FF* | 66 |
| **15** | 10-14 | DeltaF508/DeltaF508 | *MSSA* | 88 |
| **16** | 18-22 | 2789+5G/2183AA-G | *P.a.* | 119 |
| **17** | 35-39 | G542X/DeltaF508 | *P.a. MS, MDR P.a., C.a.* | 96 |
| **18** | 24-28 | N1303K/E585X | *MDR P.a., MSSA* | 67 |
| **19** | 11-15 | N1303K/N1303K | *MSSA* | 99 |
| **20** | 50-54 | DeltaF508/2789+5G->A | *S.m., P.m.* | 89 |
| **21** | 19-23 | DeltaF508/1845delAG | *MSSA* | 86 |
| **22** | 32-36 | N1303K/N1303K | *P.a.* | 71 |

**Abbreviations**. FEV-1: Forced Expiratory Volume in the 1st second; *NTM: Nontuberculous mycobacteria; FF: Filamentous fungi; P.a.: Pseudomonas aeruginosa; MS P.a.: Pseudomonas aeruginosa mucoid strain; MDR P.a.: Pseudomonas aeruginosa multi drug resistant; MRSA: Methicillin-resistant Staphylococcus aureus; A.f.: Aspergillus fumigatus; A.x.: Achromobacter xylosoxidans; MSSA: Methicillin-sensitive Staphylococcus aureus; C.a.: Candida albicans; E.c.: Escherichia coli; E.f.: Enterococcus faecalis; S.m.: Serratia marcescens; P.m.: Proteus mirabilis.* The age of each patient was reported as age range in order to protect patient privacy and prevent their identification.
